# A Reporter System for Fast Quantitative Monitoring of Type 3 Protein Secretion in Enteropathogenic *E. coli*

**DOI:** 10.3390/microorganisms8111786

**Published:** 2020-11-14

**Authors:** Luit Barkalita, Athina G. Portaliou, Maria S. Loos, Biao Yuan, Spyridoula Karamanou, Anastassios Economou

**Affiliations:** Rega Institute for Medical Research, Laboratory of Molecular Bacteriology, Department of Microbiology, Immunology and Transplantation, KU Leuven, B-3000 Leuven, Belgium; luit.barkalita@kuleuven.be (L.B.); Athina.Portaliou@KULEUVEN.BE (A.G.P.); maria.loos.be@gmail.com (M.S.L.); chinayuanbiao@163.com (B.Y.); lily.karamanou@KULEUVEN.BE (S.K.)

**Keywords:** EPEC, Type 3 secretion, quantitative in vivo secretion assay, alkaline phosphatase activity, SctA-PhoA

## Abstract

The type 3 secretion system is essential for pathogenesis of several human and animal Gram-negative bacterial pathogens. The T3SS comprises a transmembrane injectisome, providing a conduit from the bacterial cytoplasm to the host cell cytoplasm for the direct delivery of effectors (including toxins). Functional studies of T3SS commonly monitor the extracellular secretion of proteins by SDS-PAGE and western blot analysis, which are slow and semi-quantitative in nature. Here, we describe an enzymatic reporter-based quantitative and rapid in vivo assay for T3SS secretion studies in enteropathogenic *E. coli* (EPEC). The assay monitors the secretion of the fusion protein SctA-PhoA through the injectisome based on a colorimetric assay that quantifies the activity of alkaline phosphatase. We validated the usage of this reporter system by following the secretion in the absence of various injectisome components, including domains of the gatekeeper essential for T3SS function. This platform can now be used for the isolation of mutations, functional analysis and anti-virulence compound screening.

## 1. Introduction

The type three protein secretion system (T3SS) is essential for the pathogenic potential of many Gram-negative bacteria [1,2]. Pathogens including *Salmonella*, *Shigella*, *Yersinia*, *Pseudomonas* and Enteropathogenic *E. coli* (EPEC) use the T3SS to directly deliver effectors (including toxins) from their cytoplasm to that of the host cell [2,3].

**Figure 1:**
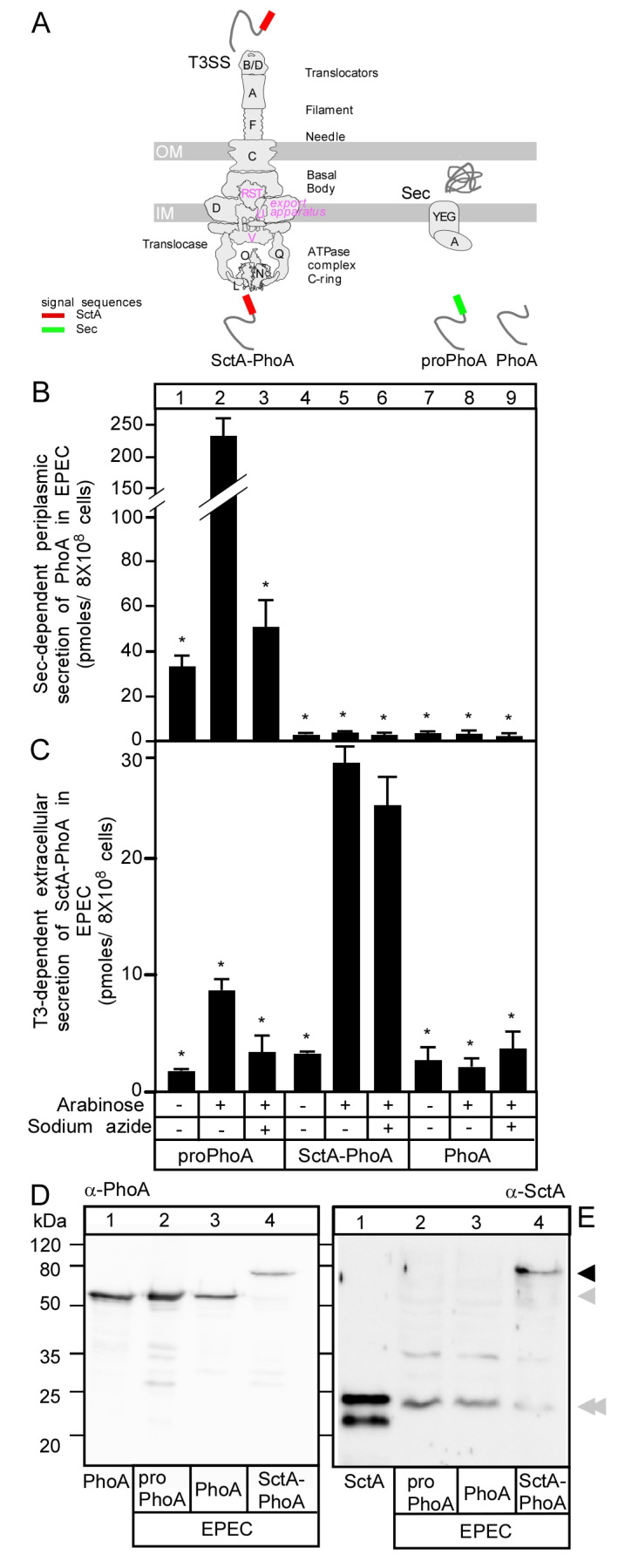
SctA-PhoA secretion by EPEC cells is not Sec-dependent. (**A**) Cartoon of the T3SS proteins forming an injectisome and a SecYEG channel. Letters indicate the protein name following the Sct nomenclature (i.e., V = SctV). Below the injectisome, the secretory choice of the indicated secretory proteins, signal sequences, and their localizations after secretion are shown. (**B**,**C**) Testing of Sec-dependent periplasmic and T3SS-dependent extracellular secretion of proPhoA, SctA-PhoA and PhoA in EPEC, respectively, as derived from PhoA enzymatic activity (see Materials and Methods). Arabinose (0.13 mM) was used to induce the production of PhoA derivatives and sodium azide (4 mM) to prevent SecA-mediated secretion, as indicated. Bar graphs with SEM are shown; *n* = 3 biological repeats. Unpaired parametric *t*-test was performed, *: *p* < 0.01. (**D**,**E**) Intracellular production of proPhoA, PhoA and SctA-PhoA in EPEC. Polypeptides were analyzed in 15% *w*/*v* acrylamide gels by SDS-PAGE, followed by immunostaining with α-PhoA and α-SctA (as indicated). Arrows indicated: SctA-PhoA (black); PhoA (grey); chromosomal SctA (double grey). Lanes 1: 100 ng of PhoA-His (**D**) and 100 ng SctA (**E**) purified as described [15,16], respectively. A representative image is shown; *n* = 3 biological repeats.

At its core, the system comprises a multiprotein injectisome that forms a conduit bridging the bacterial and the host cell plasma membranes [1,4]. The injectisome is divided into three parts: (i) the cytoplasmic part, composed of the ATPase complex and its regulators, which peripherally associate with the inner membrane embedded translocase or export apparatus [5], (ii) the basal body, which contains stacks of inner and outer membrane rings, which encircle the inner rod and contribute to the formation of a tubular conduit through the periplasmic space linking the export apparatus at one end and the external needle (iii) at the other [6,7]. The needle tip contains the translocon complex, which physically contacts the host plasma membrane (Figure 1A) [1,8]. Injectisome assembly and the secretion caused by it are finely regulated [1,9]. Assembly begins with the Sec system-dependent secretion of the inner (SctD and J) and outer (SctC) membrane ring components, which build the outer shell of the basal body [10,11]. In parallel, the multimeric export apparatus components (SctRSTU and V) are integrated in the inner membrane through the Sec system and are encircled by the inner membrane ring components SctD and SctJ [12]. Once the cytoplasmic ATPase (SctN), in complex with its auxiliary factors (SctL, O, Q), peripherally associate with the export apparatus, the partially completed injectisome becomes competent for T3S [13]. The first group of proteins secreted are the early substrates comprising the needle length regulatory protein (SctP), the inner rod protein (SctI) and the needle protein (SctF) [4] to form the extracellular structural elements of the injectisome. Then, the secretion switches to that of middle substrates [14] comprising the filament protein (SctA) and the translocon components (SctB and E) [3,9] that complete the assembly of the injectisome (Figure 1A). This enables the secretion/injection of the late T3S substrates (effectors) in response to specific environmental stimuli [9].

Here, we use the EPEC T3SS model, which is responsible for diarrheal diseases in humans and animals [17,18]. During EPEC pathogenesis in the gut, fully assembled injectisomes attach to the host enterocyte plasma membrane, to which the translocon becomes embedded [19]. This enables EPEC to sense the host cytoplasmic environment, which is low in Ca^2+^ (~120 nM) compared to that in the gut (mM range). This high to low Ca^2+^ concentration shift signals the secretion of effectors by an unknown mechanism [20]. In one model, the gatekeeper protein SctW forms a Ca^2+^-sensitive complex with the molecular ruler SctP. This complex, which is stable in high Ca^2+^, presumably promotes the secretion of middle substrates (i.e., the translocator proteins) and blocks the secretion of late ones (i.e., the effectors). Once the translocator pore is formed, the concentration of Ca^2+^ drops and the SctP-SctW complex gets dissociated; thus, late substrates can be secreted [21].

Monitoring T3S using a robust, quick and quantitative assay is essential for studying the function of T3SS components and secretion regulation. Many attempts used different chimeric proteins composed of full length or truncated T3 substrates fused to an heterologous protein (i.e., an enzyme) and secretion through T3SS was monitored [22,23] either by immunostaining [22,24] or fluorescence microscopy [22] or spectroscopically [23]. Adenylate cyclase was N-terminally fused to effectors to monitor their translocation into HeLa cells by measuring cAMP level [25,26]. β-lactamase, the catalytic domain of phospholipase A2, or Alkaline Phosphatase (PhoA) [27] was fused to different T3 substrates to report on protein secretion into eukaryotic cells [28,29,30]. Recently, the development of a light-dependent T3SS-mediated heterologous fluorescent protein delivery into eukaryotic cells was reported [31]. These methods are more quantitative and faster than secreted protein detection using SDS-PAGE analysis followed by Coomassie and/or immuno-staining [3,9,32,33].

Here, we developed a reporter-fusion assay based on PhoA activity to monitor in vivo translocator secretion through the injectisome of EPEC in spent growth medium of bacterial cultures [15,34]. Wild-type PhoA in the bacterial cytoplasm is expressed as a pre-protein (proPhoA), which contains a 20 amino acid-long N-terminal signal peptide and a mature domain (PhoA) (Figure 1A) [15]. The signal peptide is essential for the Sec-mediated translocation of PhoA and is cleaved upon PhoA release to the periplasm [35]. There, PhoA dimerizes, forms disulfides, binds metals and converts into the active enzyme [35]. In the absence of its signal peptide, PhoA cannot be translocated to the periplasm and remains inactive and unfolded in the cytoplasm (Figure 1A, right) [36]. Unlike Sec substrates, the ones of the T3SS contain a non-conserved, non-cleavable secretion signal usually within the first 20 amino acids [32,37] making it part of the secreted protein [32,38]. Here, we replaced the N-terminal Sec signal peptide of PhoA with the T3SS translocator SctA [2,39] to generate the chimeric protein SctA-PhoA. Upon secretion, the SctA anchors on the needle of the injectisome and self-polymerizes to form filaments that can be 2 µm long. SctA secretion is essential for the adhesion of EPEC to eukaryotic cells and thus for pathogenesis [9,16,39,40]. Due to the lack of the Sec-signal sequence, SctA-PhoA is not recognized by the Sec translocase but is recognized by the T3S injectisome and secreted to the extracellular milieu to form an active PhoA that can be monitored enzymatically (Figure 1A, left).

This system was developed and validated by testing for the function of T3SS components, secretion hierarchy and Ca^2+^-mediated regulation [41] and provides a platform for future functional studies and drug discovery.

## 2. Materials and Methods

For the complete list of strains, plasmids, mutants, primers, buffers and antibodies, see the Supplementary Material.

### 2.1. Generation of the sctA-phoA Construct

The *sctA* gene (Uniprot accession number B7UM94) was amplified from the wild type EPEC (E2348/69) and inserted so as to encode an N-terminal fusion in pBAD501PhoA (Table S5) [42], substituting the native PhoA (Uniprot accession number P00634) signal sequence, after NdeI-HindIII digestion (primers listed in Table S6). The new construct was verified by sequencing (Macrogen, Amsterdam, The Netherlands). For the exact protein sequence, see the Supplementary Material.

### 2.2. Optimization of Media

The M9 medium previously optimized for T3SS secretion by EPEC (M9-mod1; [43]) was further modified in order to grow cells that could be assayed by the alkaline phosphatase activity (M9-mod2; Table S1). Briefly, phosphate buffer was replaced by 50 mM HEPES (pH 7.6) so as to avoid competition with PhoA activity. Glucose that represses the pAra promoter was replaced by glycerol (0.4% *v*/*v*) [44]. To increase the bacterial growth rate (OD_600_ = 0.3; 3 h; 37 °C; shaking incubator at 180 rpm), M9-mod2 medium was also supplemented with 0.4% *w*/*v* casamino acids (instead of 0.2% *w*/*v*, as used in M9-mod1).

### 2.3. Bacterial Culture for In Vivo Secretion Assay

EPEC or derivative cells were made competent to accept foreign DNA by a chemical method [45] that was transformed following a standard transformation protocol [45] (45 s at 42 °C, 2 min on ice, 1 h recovery at 37 °C) with the pBAD501*sctA-phoA* plasmid (pAra promoter). Single colonies were used to inoculate 5 mL of Luria-Bertani (LB) broth in a 15 mL glass test tube and bacterial cultures were grown aerobically in a shaking incubator (37 °C; 15 h; 180 rpm) and used as inoculum (1:50 dilution; 15 mL; in glass test tubes) in freshly prepared M9-mod2. After a 3 h incubation (or until OD_600_ ~ 0.3) under the same conditions. SctA-PhoA production was induced by freshly prepared arabinose (0.13 mM; 3 h; 37 °C).

In the case of cells carrying an additional pASK-IBA7^+^ vector (pTet promoter) with genes encoding for chaperone or gatekeeper derivatives, gene expression was first induced for 30 min with anhydrotetracycline (AHT; 5 ng/mL). Then, the production of SctA-PhoA was induced as above.

### 2.4. In Vivo Alkaline Phosphatase Assay

Following the induction of SctA-PhoA production, 1 mL of culture was transferred into a 1.5 mL microcentrifuge tube and centrifuged (1500× *g*; 8 min; 4 °C). From that, 500 µL of supernatant was transferred into a fresh 1.5 mL microcentrifuge tube (spent growth medium samples) and 0.5 mL of the remaining supernatant was removed without disturbing the cell pellet.

#### 2.4.1. Treatment of the Spent Growth Medium Samples

To 500 µL of culture supernatant, 0.05 N NaOH (final concentration) was added to bring the pH to 8.0, which is optimal for PhoA activity. Then, 10 mM (final concentration) of para-nitrophenyl phosphate from 1 M stock solution in dH_2_O (PNPP; Thermo Scientific, Waltham, MA, USA; stored at −20 °C) was added and samples were incubated at 37 °C (pre-warmed water bath) for approximately 10 min (until a light-yellow color developed). In parallel, to determine PNPP self-hydrolysis, 10 mM of PNPP (final concentration) was added in 500 µL of 1 M Tris (pH 8.0), which was incubated with the samples. To terminate PNPP hydrolysis, a final concentration of 16.67 mM K_2_HPO_4_ was added and samples were transferred on ice for 10 min after vortexing briefly. To determine PNPP hydrolysis, 250 µL from each tube was transferred to a microtiter plate and OD values were measured spectrophotometrically (iControl; TECAN-infinite M200, Tecan Trading AG, Männedorf, Switzerland) at 420 nm. OD values from all samples were normalized by subtracting the one determined for self-hydrolysis.

#### 2.4.2. Treatment of the Cell Samples

The cell pellet derived from 1 mL culture was resuspended in 1 mL of 1 M Tris (pH 8.0) buffer. Then, to further dilute the samples, 100 µL of the cell suspension was added to 900 µL of 1 M Tris (pH 8.0) buffer in a fresh tube (1:10 dilution). From that diluted cell suspension, 500 µL were transferred to a fresh 1.5 mL microcentrifuge tube, 10 mM (final concentration) of PNPP was added and samples were incubated at 37 °C (pre-warmed water bath) for approximately 10 min (until a light-yellow color developed). In parallel, PNPP self-hydrolysis was determined as above. To terminate the PNPP reaction, samples were supplemented with a final concentration of 16.67 mM K_2_HPO_4_ and 0.17% *v*/*v* Triton-X-100 (ACROS) and transferred on ice for 10 min after vortexing briefly. Cells and debris were pelleted by centrifugation (15,000× *g*; 5 min; 4 °C) and 250 µL supernatant from each tube was measured in a microtiter plate at OD_420_ (hydrolyzed PNPP absorbance). To calculate the PhoA activity per cell, we determined the optical densities of the bacterial culture by measuring the OD values of the cell suspensions at 600 nm. To do so, 250 µL of the final cell suspension and the initial undiluted one were used. The same volume of 1 M Tris (pH 8.0) buffer served as a blank. OD values were measured spectrophotometrically (iControl; TECAN-infinite M200, Tecan Trading AG, Männedorf, Switzerland).

The OD measurements obtained above were used for the phosphatase activity of PhoA determination [15] using the following formula:

Units of PhoA activity = OD_420_ × 1000 × Dilution factor/(OD_600_ × time_min_) (1)

Dilution factor for supernatant = 1.21(2)

Dilution factor for cells = 1.22(3)

Phosphatase activity was extrapolated to secreted PhoA amounts based on a standard curve of PhoA enzymatic activity plotted as a function of protein concentration (Figure S1A).

## 3. Results

### 3.1. Secretion of SctA-PhoA Is T3SS-Dependent

First, we compared the secretion of proPhoA (Sec substrate), SctA-PhoA (potential T3SS substrate) and PhoA (non-Sec and non-T3S secreted) (Figure 1B,C). All three proteins were stably produced in EPEC intracellularly as confirmed by immunostaining using anti-PhoA and anti-SctA antibodies (Figure 1D,E, respectively), but their secretion properties differed. The secreted amounts of SctA-PhoA were quantified after western blot analysis using standard amounts of purified PhoA (Figure S1B,C). In addition, the amounts of secreted proPhoA or SctA-PhoA were further quantified using a standard curve (Figure S1A) of PhoA enzymatic activity and were found to be similar. Therefore, the two quantification methods were in good agreement (no significant difference in *t*-test) between them (Figure S1D).

Upon induction of synthesis, proPhoA was secreted into the periplasmic space of EPEC (Figure 1B, lane 2) or BL21 (Figure S2) cells. Its secretion was inhibited by the Sec-system inhibitor sodium azide (Figure 1B, lane 3), consistent with its secretion being Sec pathway-dependent. On the other hand, when its synthesis was induced, SctA-PhoA was secreted extracellularly into the spent medium of EPEC (Figure 1C, lane 4 vs. 5) cells and acquired measurable enzymatic activity that was not inhibited by sodium azide (Figure 1C, lane 6). Moreover, PhoA activity was not detected in the cell fraction (Figure 1B, lane 5) or in the supernatant of BL21 cells, which do not carry a T3SS (Figure S2). Collectively, these data suggested that the observed secretion is not Sec-dependent but rather requires the T3SS. Native SctA does not interfere with SctA-PhoA secretion EPEC (Figure S1E). PhoA is secreted neither through the Sec (Figure 1B, lanes 8 and 9) nor through the T3S (Figure 1C, lanes 8 and 9) system, confirming the importance of a guiding signal.

**Figure 2:**
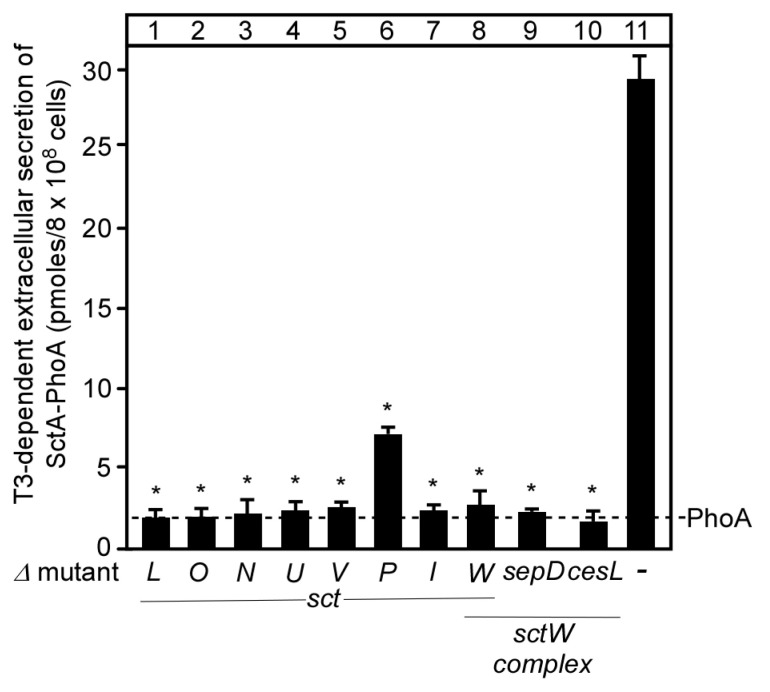
SctA-PhoA requires T3SS components for secretion. Quantification of SctA-PhoA secreted in the spent growth medium (as in Figure 1C) in different EPEC gene-deletion strains. Letters indicated the deleted gene (as in Figure 1A). Bar graphs with SEM are shown; *n* = 3 biological repeats. Unpaired parametric *t*-test was performed, *: *p* < 0.01.

Therefore, SctA-PhoA displayed apparent a T3SS-dependent secretion in EPEC. To directly test this, we monitored its extracellular secretion (Figure 2) in EPEC mutant derivatives, carrying different deletions of genes encoding components of the ATPase complex (Δ*sctL*, Δ*sctO*, Δ*sctN*), the export apparatus (Δ*sctU*, Δ*sctV*), the needle length regulator *(*Δ*sctP*), the inner rod (Δ*sctI*) and the gatekeeper switch complex (Δ*sctW*, Δ*sepD* and Δ*cesL*). These genes are all essential or important for SctA secretion through the injectisome [1,46]. SctA-PhoA was synthesized in all derivatives (Figure S3, lanes 2–11), but none of them yielded any detectable extracellular secretion (lanes 1–5 and 7–10), except EPECΔ*sctP* (lane 6), which showed that secretion was reduced to ~28%. These results are consistent with genetic [34] and immuno-staining [3,34] analyses and validated the applicability of the SctA-PhoA reporter system to monitor the function of T3SS components.

### 3.2. T3S-Dependent Secretion of SctA-PhoA and Native SctA Have the Same Requirements

**Figure 3:**
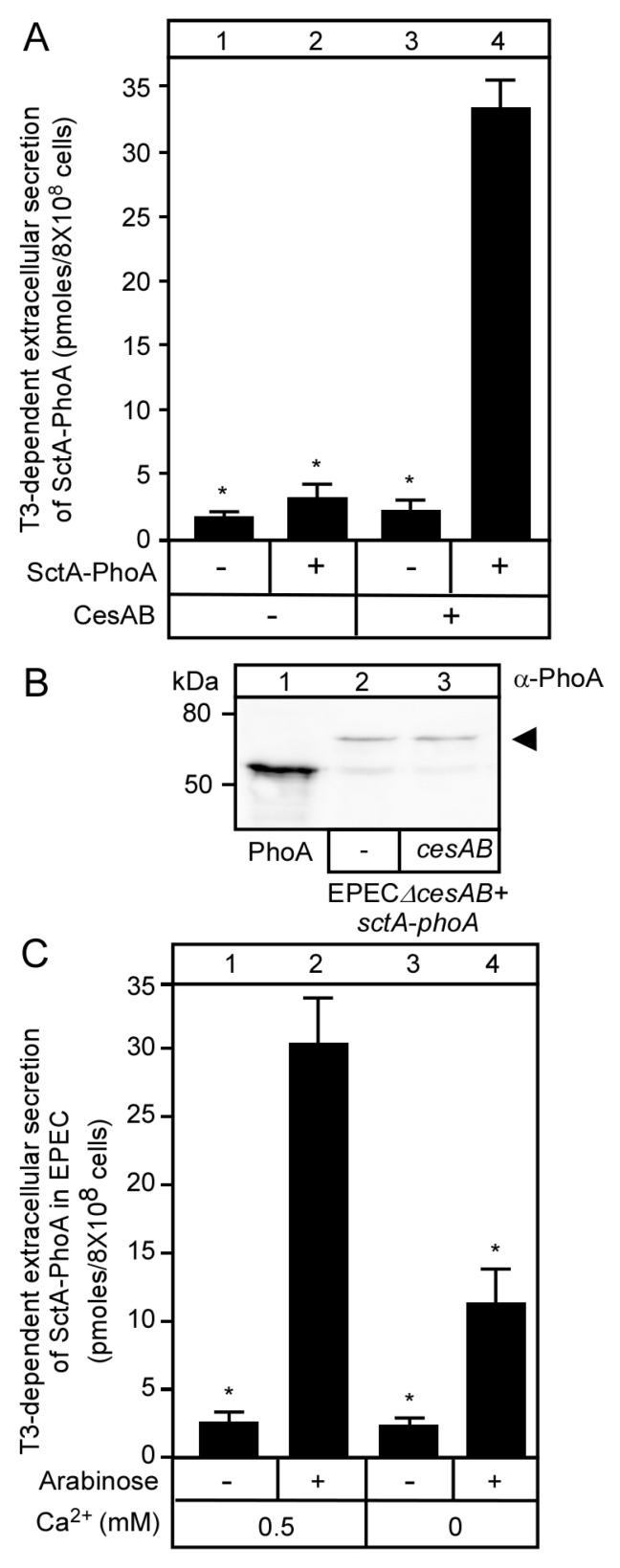
T3S-dependent secretion of SctA-PhoA and native SctA have the same requirements. (**A**) Quantification of SctA-PhoA secreted in the absence and presence of the CesAB chaperone in EPECΔ*cesAB* carrying pASK-IBA7^+^*cesAB*; Bar graphs with SEM are shown; *n* = 3 biological repeats. Unpaired parametric *t*-test was performed, *: *p* < 0.01. (**B**) Intracellular production of SctA-PhoA in the absence or presence of CesAB in EPECΔ*cesAB* cells. Polypeptides were analyzed as in Figure 1D, and immuno-stained with α-PhoA. A representative image is shown. Arrow indicates SctA-PhoA; Lane 1: 100 ng of PhoA-His purified as previously described [15]. *n* = 3 biological repeats. (**C**) Quantification of SctA-PhoA secreted extracellularly by EPEC cells in the presence (0.5 mM CaCl_2_) and absence (0.1 mM EGTA) of Ca^2+^. Bar graphs with SEM are shown; *n* = 3 biological repeats. Unpaired parametric *t*-test was performed, *: *p* < 0.01.

The CesAB chaperone stabilizes SctA in the cytoplasm [39,47] and targets it to the membrane by virtue of its affinity to the membrane-bound T3S translocase-associated SctW complex [9]. We, therefore, examined whether CesAB, similar to native SctA, is necessary for the stability and secretion of SctA-PhoA. For this, EPECΔ*cesAB* cells carrying pBAD501-*sctA-phoA* were additionally transformed with pASK-IBA7^+^ without or with *cesAB*, expressed under a tetracycline promoter. Extracellular secretion of SctA-PhoA was observed in the presence (Figure 3A, lane 4) but not in the absence (lane 2) of CesAB, although α-PhoA immunostaining of whole cell extracts revealed that SctA-PhoA was stably synthesized in both cases (Figure 3B, lanes 2 and 3). These findings were comparable to the immunodetection of SctA in spent growth medium supernatants, although in the absence of CesAB, no SctA was detected in the cell extracts [47], suggesting that the PhoA moiety may additionally stabilize SctA-PhoA in the absence of CesAB.

We next examined the dependence of SctA-PhoA secretion on Ca^2+^. Similar to native SctA (Figure S4), SctA-PhoA was secreted 2-fold more in the presence (Figure 3C, lane 2) of mM concentrations of Ca^2+^ than in their absence (lane 4). We concluded that SctA-PhoA is secreted indistinguishably from SctA, and therefore, the PhoA moiety does not interfere with the T3S substrate switching process.

### 3.3. Functional Characterization of SctW Mutants Using the SctA-PhoA Reporter

**Figure 4:**
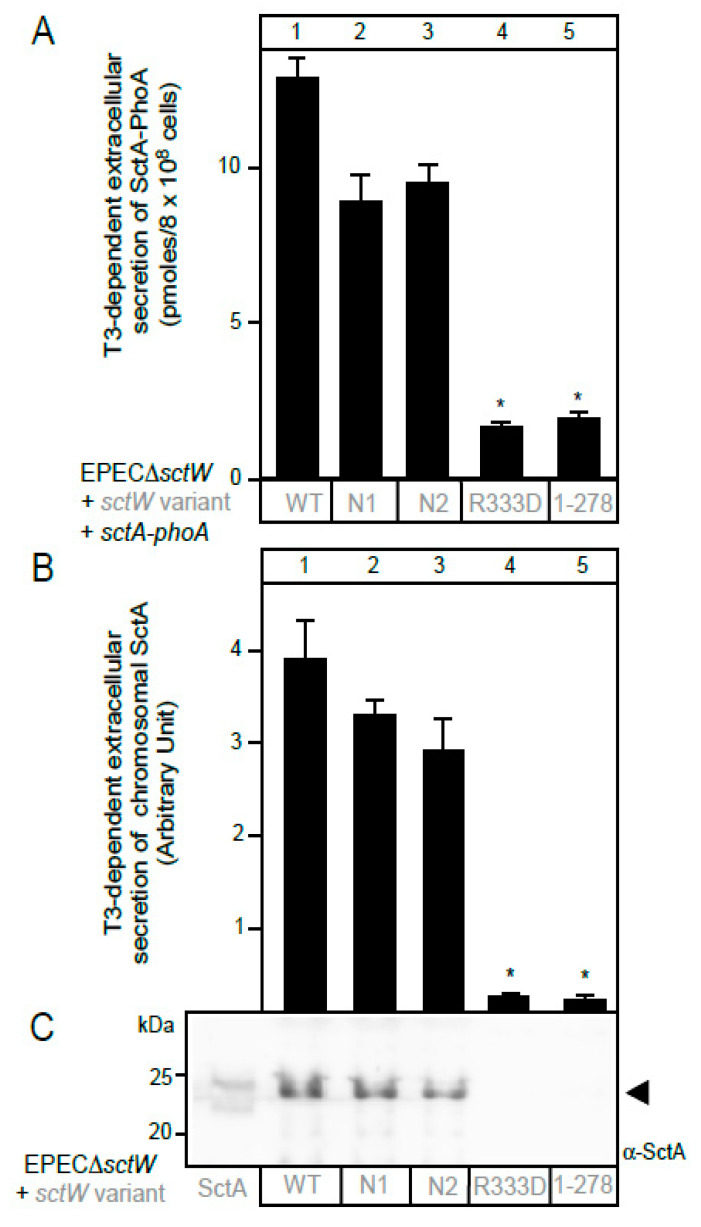

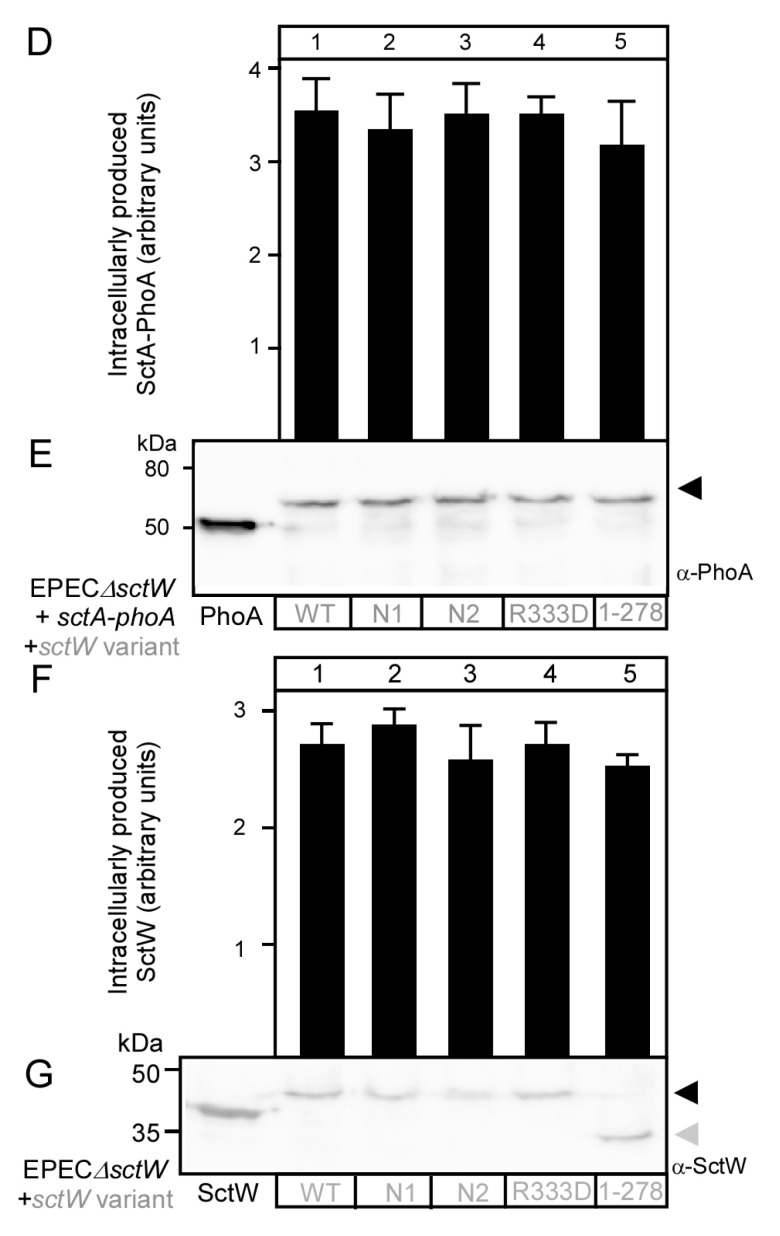
Functional characterization of *sctW* mutants. (**A**) Quantification of SctA-PhoA secreted extracellularly from EPECΔ*sctW* cells complemented with *sctW* or mutant variants (as indicated) carried on the pASK-IBA7^+^ vector. Bar graphs with SEM are shown; *n* = 3 biological repeats. Unpaired parametric *t*-test was performed, *: *p* < 0.01. (**B**) Quantification of chromosomal SctA secreted extracellularly from EPECΔ*sctW* cells complemented with *sctW* or mutant variants. Signal intensities were quantified using Image J software (Schneider et al., 2012) and are shown in bar graphs with SEM; *n* = 3 biological repeats. *: *p* < 0.01. (**C**) Image of a representative western blot analyzing extracellularly secreted SctA in EPECΔ*sctW* cells complemented with *sctW* or mutant variants (as indicated). Polypeptides were analyzed as in Figure 1D, and immuno-stained with α-SctA. Left: 50 ng of SctA, purified as previously described [16]. The arrow indicates SctA. A representative image is shown; *n* = 3 biological repeats. (**D**) Quantification of inracellularly produced SctA-PhoA (as in **B**). Bar graphs with SEM are shown. *n* = 3 biological repeats. (**E**) Intracellular production of SctA-PhoA in EPECΔ*sctW* cells carrying *sctW* or derivatives (as indicated). Polypeptides were analyzed as in Figure 1D. Left: 100 ng of PhoA-His purified as previously described [15]. Black arrows: SctA-PhoA. A representative image is shown; *n* = 3 biological repeats. (**F**) Quantification of SctW signal intensities (as in **B**). Bar graphs with SEM are shown; *n* = 3 biological repeats. (**G**) Intracellular production of SctW or of the indicated mutant derivatives. Polypeptides are analyzed as in Figure 1D, and immuno-stained with α-SctW. Left: 100 ng of SctW purified as described previously [48]. Arrows: SctW and SctW (R333D) (black) and truncated SctW (gray). A representative image is shown; *n* = 3 biological repeats.

The SctW gatekeeper is a translocase-associated receptor for middle substrates/chaperones and switches affinity in the EPEC T3SS from middle substrates to effectors [9]. It has an unstructured N-terminal region that is responsible for membrane localization and for binding of chaperones SepD and CesL and a three-domain main body [48]. Membrane-bound SctW interacts with the cytoplasmic domain of the major export apparatus protein SctV [9]. To test the applicability of our assay in characterizing SctW-mediated SctA secretion, we generated four derivatives of SctW (Table S1). Two derivatives (N1 and N2) are poly-alanine substitution mutants in the N-terminal disordered region of SctW for increasing the helical propensity [49] of the N-terminal domain; one had a carboxy-terminal point mutation (R333D) at a conserved residue that is important for middle substrate/chaperone binding in *Chlamydia* [48] and a C-terminal domain truncation [SctW(N1-278)]. The effect of these mutations on SctA-PhoA secretion was tested. For this, EPECΔ*sctW* cells transformed with pBAD501-*sctA-phoA* were additionally transformed with variants of *sctW* cloned in vector pASK-IBA7^+^, under a tetracycline promoter [50], and the effect of the SctW mutations on the extracellular secretion of SctA-PhoA was compared to that of SctW (Figure 4A). All recombinant proteins were shown by immuno-staining to be synthesized (Figure 4B,C). While secretion driven by the N-terminal mutants was modestly compromised (~25%; Figure 4A lanes 2 and 3), that of the C-terminal mutants was abrogated (lanes 4 and 5).

## 4. Discussion

T3SSs are being studied to better understand the pathobiology of diseases and to develop anti-virulence pharmaceuticals and protein delivery systems. Monitoring secretion through the injectisome in vivo is fundamental to studying the system’s functionality.

Here, we developed a rapid quantitative assay of secretion of the chimeric SctA-PhoA protein in EPEC cells though the T3SS (Figure 1A). SctA-PhoA secretion made use of the T3SS machinery and was at similar levels as those of wild type SctA (Figure 2 and Figure 4) [3,13,51,52,53]. This assay is simplified by monitoring extracellular SctA-PhoA secretion in the spent growth medium of a bacterial culture, in the absence of eukaryotic cells. The similar secretion pattern of SctA-PhoA (Figure 4A) and that of chromosomally encoded SctA (Figure 4B) in EPECΔ*sctW* cells that were complemented with SctW variants validates the use of SctA-PhoA secretion as a reporter of native SctA secretion. Additionally, as SctA secretion is essential for T3SS-mediated injection of effectors during infection [16,54,55,56], this assay is applicable to functional studies of any injectisome component.

SctA-PhoA secretion required the CesAB chaperone that is essential for SctA secretion [47,57] (Figure 3A). However, unlike native SctA, SctA-PhoA was stably expressed in EPEC in the absence of its chaperone (Figure 3B). Presumably, the C-terminal PhoA stabilizes the N-terminal SctA moiety in the cytoplasm, suggesting long-term conformational effects in the chimeric molecule, perhaps facilitated by the non-folded nature of reduced PhoA [42,58]. The failure of the SctA-PhoA to get secreted in EPECΔ*cesAB* cells indicated that even though CesAB was not required for cytoplasmic stability, it was still essential for secretion. Presumably, this is due to its requirement for enhanced solubility and targeting it to the SctW complex on the translocase [9]. We assume that nascent SctA-PhoA may interact with CesAB before translation of the C-terminal PhoA is even complete. Such a mechanism would quickly sort T3SS substrates away from Sec pathway proteins and cytoplasmic residents. Similarly, the chimeric SptP-PhoA is stable without its chaperone SicP, which is essential for the stability of the native SptP [27].

Based on the above, C-terminally fused PhoA did not interfere with the SctA moiety, which was properly recognized and secreted as a T3SS substrate. These findings agree with previous studies where different T3SS substrates (in full or in part) N-terminally fused to different tags [32,34]. This suggested that the significantly unstructured T3SS clients retain their conformational disorder and targeting signal exposure, and thus, would be structurally independent from the C-terminal PhoA. This is expected to allow multiple cargo proteins to be carried out in biomedical or biotechnological applications [59]. Moreover, the length of the exported molecules is apparently not an obstacle for efficient secretion and suggests that while the injectisome may use a molecular ruler type mechanism to control its length, this need not be relevant for subsequent client export.

Secretion through T3SS is well-orchestrated and hierarchical [2]. SctW and SctV function as a bipartite membrane receptor for CesAB:SctA [9]. The N-terminal disordered region of SctW is necessary for membrane anchoring and for binding of the SctW chaperones [9,48]. These regions are important but not essential for SctA-PhoA secretion (Figure 4). In contrast, the SctW C-terminal region, while not important for docking to the SctW/SctV receptor [9], is essential for secretion (Figure 4) and switching [9]. The C-terminal region also interacts with a different class of middle client chaperone (SctE/CesD) in the homologous system of *Chlamydia* [60]. We presume that the allosteric effect of SctW on SctV is being compromised as the C-terminal end of SctW contributes to interactions with SctV [9].

The availability of the SctA-PhoA system with its quantitative nature and rapid responses now allows us to systematically isolate random mutant derivatives on SctW and SctV, to identify functionally important sites and locked conformational states using plates for screens with the substrate XP [61]. In vivo, a functional T3SS delivers effectors to the host cell [19]. The assay developed here monitors only T3SS function based on secretion into the medium and not the actual translocation/injection into host cell.

As we have transferred the PhoA detection assay to a high throughput-screening format using luminescence detection [62], the platform described here is now available to specifically screen for anti-virulent inhibitors.

## Supplementary Materials

The following are available online at https://www.mdpi.com/2076-2607/8/11/1786/s1, Figure S1: Characterization of SctA-PhoA activity (Related to Figure 1). Figure S2: Sec-dependent periplasmic secretion of proPhoA and SctA-PhoA in *E. coli* BL21 (Related to Figure 1). Figure S3: Intracellular production of SctA-PhoA in different EPEC knock-out strains (Related to Figure 2). Figure S4: Secretion of SctA in EPEC in absence and presence of Ca^2+^ (Related to Figure 3), Table S1: Genetic constructs.
