# Supplementary material for: A Reporter System for Fast Quantitative Monitoring of Type 3 Protein Secretion in Enteropathogenic E. coli
Source: Microorganisms. 2020 Nov 14;8(11):1786. doi: 10.3390/microorganisms8111786 (PMC7696366; doi:10.3390/microorganisms8111786)
Supplement: Supplementary file 1 [file microorganisms-08-01786-s001.pdf]

A Reporter System for Fast Quantitative Monitoring of Type 3 Protein Secretion in Enteropathogenic *E. coli*

Luit Barkalita, Athina G. Portaliou, Maria S. Loos, Biao Yuan, Spyridoula Karamanou and Anastassios Economou*

KU Leuven, Department of Microbiology, Immunology and Transplantation, Rega Institute for Medical Research, Laboratory of Molecular Bacteriology, B-3000 Leuven, Belgium

Abbreviations

EPEC: Enteropathogenic *E. coli*
T3S: Type 3 Secretion
T3SS: Type 3 Secretion System
AHT: Anhydrotetracycline
TCA: Trichloro-acetic Acid
PNPP: Para-nitrophenyl phosphate
EGTA: Ethylene glycol-bis (β-aminoethyl ether)-N,N,N′,N′-tetra-acetic acid
OD: Optical density

Table of contents

Supplementary figures

Fig. S1: Characterization of SctA-PhoA activity (Related to Fig. 1)
Fig. S2: Sec-dependent periplasmic secretion of proPhoA and SctA-PhoA in *E. coli* BL21 (Related to Fig.1)
Fig. S3: Intracellular production of SctA-PhoA in different EPEC knock-out strains (Related to Fig. 2)
Fig. S4: Secretion of SctA in EPEC in absence and presence of Ca^{2+} (Related to Fig. 3)

Supplementary Tables

Table S1: Genetic constructs

Supplementary materials

§ Media
§ Antisera
§ Bacterial strains
§ Vectors
§ List of primers
§ Sequence of SctA-PoA

References

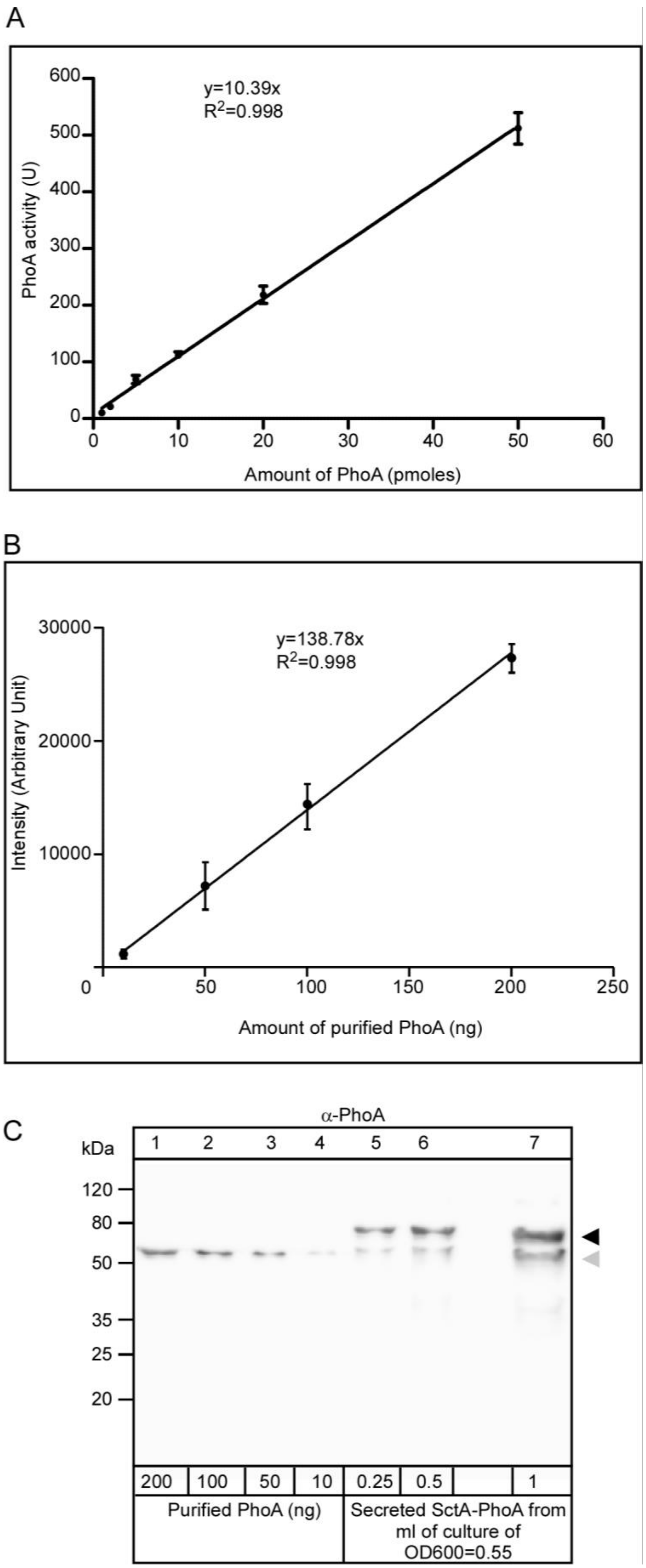
A
y=10.39x
R2=0.998
PhoA activity (U)
600
500
400
300
200
100
0
0 10 20 30 40 50 60
Amount of PhoA (pmoles)
B
y=138.78x
R2=0.998
Intensity (Arbitrary Unit)
30000
20000
10000
0 50 100 150 200 250
Amount of purified PhoA (ng)
C
α-PhoA
kDa
1 2 3 4 5 6 7
120
80
50
35
25
20
200 100 50 10 0.25 0.5 1
Purified PhoA (ng)
Secreted SctA-PhoA from ml of culture of OD600=0.55

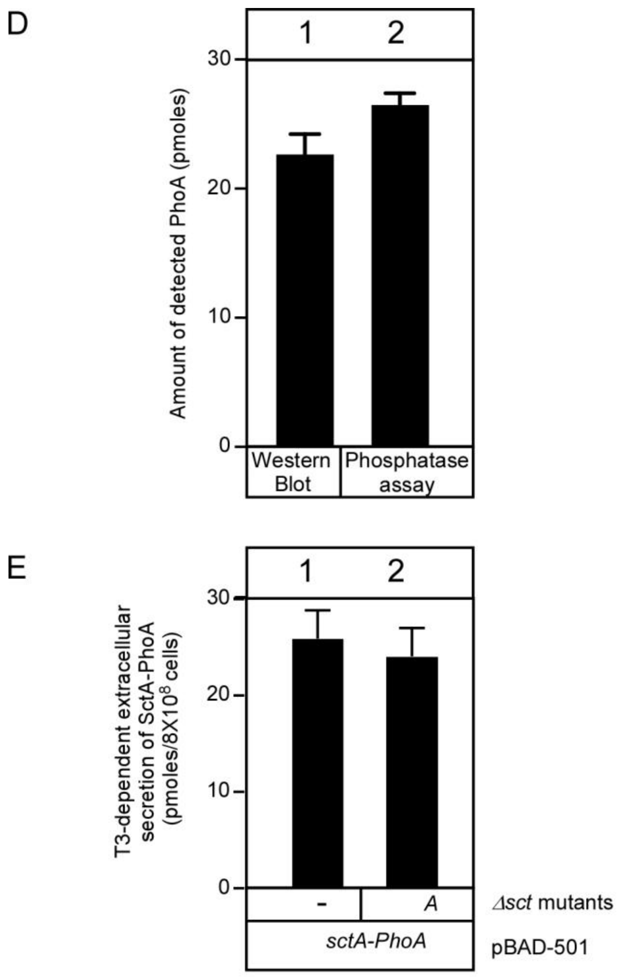

Fig. S1: Characterization of SctA-PhoA activity (Related to Fig. 1)

A) Determination of PhoA phosphatase activity

PhoA phosphatase activity was determined using different amounts of purified PhoA. For each concentration point the average value of the activity (as derived from triplicates) is used. Activity units were plotted as a function of protein concentration. Function equation was calculated from scatter plot using Microsoft Excel; *n = 3* biological repeats.

B) Generation of a standard curve of amount of PhoA vs densitometric intensity

Intensity of different amounts of purified PhoA was determined using scanning densitometry on an Image Quant LAS 4000 biomolecular imager (GE Healthcare Life Sciences) instrument and data were analyzed by Image J software version 1.8.0_172 (Schneider *et al.,* 2012). The arbitrary units of intensity were plotted as a function of protein concentration. A function equation was calculated from the scatter plot using Microsoft Excel; *n = 3* biological repeats.

C) Quantification of SctA-PhoA

Secreted SctA-PhoA was quantified by using the standard curve of Fig S1.B. A representative immunoblot is shown. The arrows on the right are indicating SctA-PhoA and PhoA (lower grey); *n = 3* biological repeats.

D) Detection of secreted SctA-PhoA by western blot and phosphatase assay

The amounts of secreted SctA-PhoA detected by western blot immuno-staining and a phosphatase assay. *n = 3* biological repeats; Unpaired parametric *t*-test was performed, *: p<0.01

E) Extracellular secretion of SctA-PhoA in EPEC or EPECΔ*sctA*

SctA-PhoA secretion is not affected from endogenous SctA. Quantification of the amount of extracellularly secreted SctA-PhoA in EPEC and EPECΔ*sctA;* Bar graphs with SEM are shown; *n = 3* biological repeats.

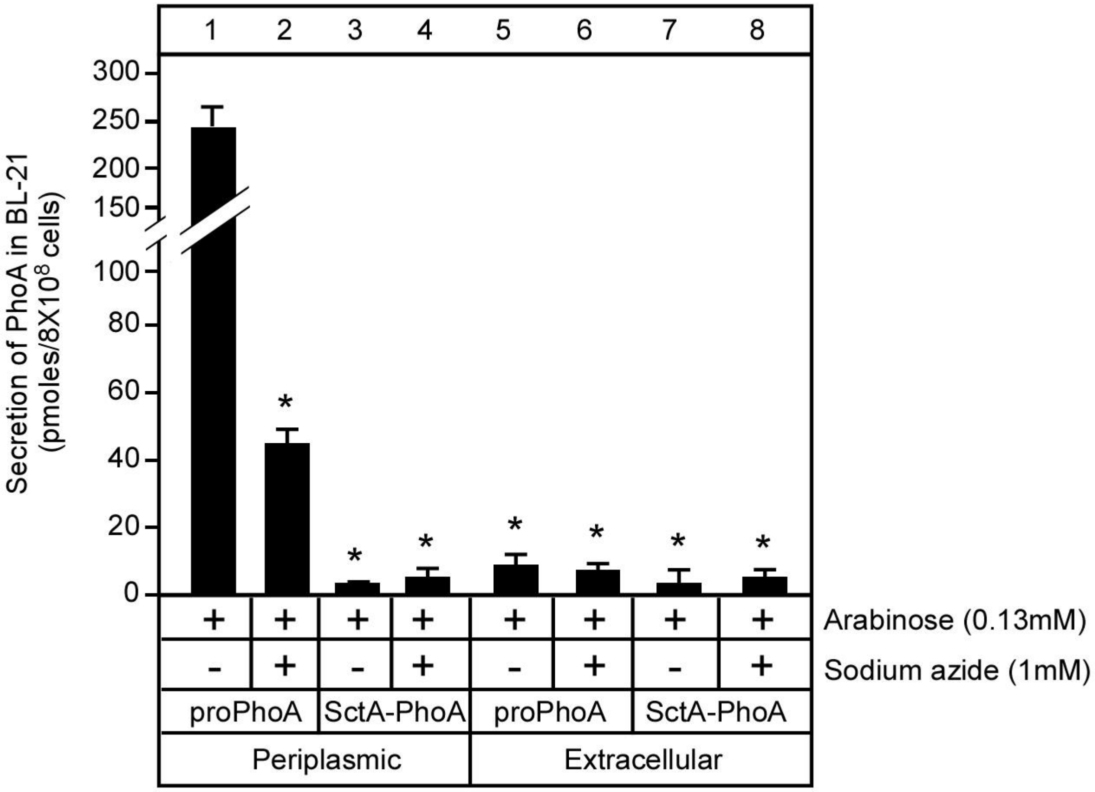

Fig. S2 Sec-dependent periplasmic and extracellular secretion of proPhoA and SctA-PhoA in *E. coli* BL21 (Related to Fig.1)

Quantification of proPhoA and SctA-PhoA amounts secreted in the periplasm and supernatant spent medium in *E. coli* BL-21 (no T3SS). PhoA phoshatase activity derived from over-synthesized proPhoA and SctA-PhoA (as indicated) in intact BL21 cells, like Fig. 2B (see Materials and Methods). Bar graphs with SEM are shown; *n = 3* biological repeats; Unpaired parametric t-test was performed, *: p<0.01

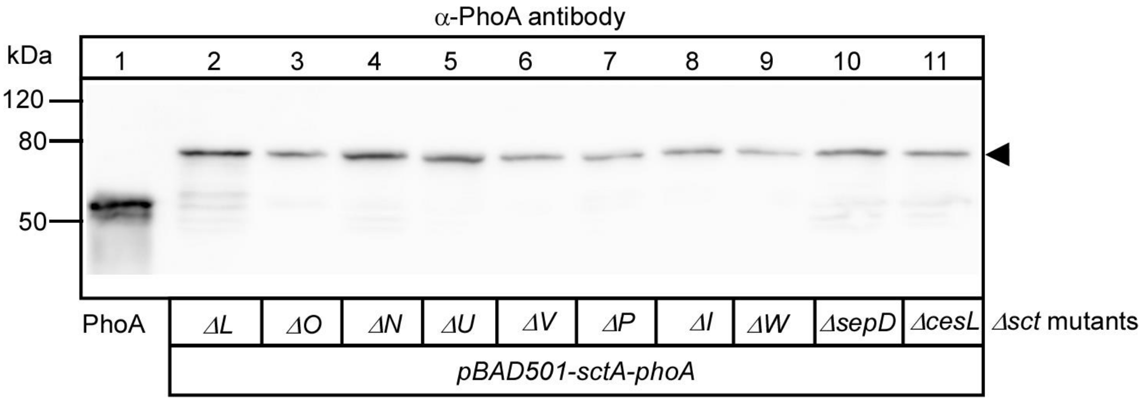

Fig. S3: Intracellular production of SctA-PhoA in different EPEC knock-out strains (Related to Fig. 2)

Intracellular production and stability of SctA-PhoA is not affected by the deletion of genes encode for different T3SS components that are essential for SctA secretion. EPEC cells wt or derivatives (as indicated) were analyzed in SDS-PAGE gel and immuno-stained using antibodies against PhoA. A representative experiment is shown; $n = 3$ biological repeats.

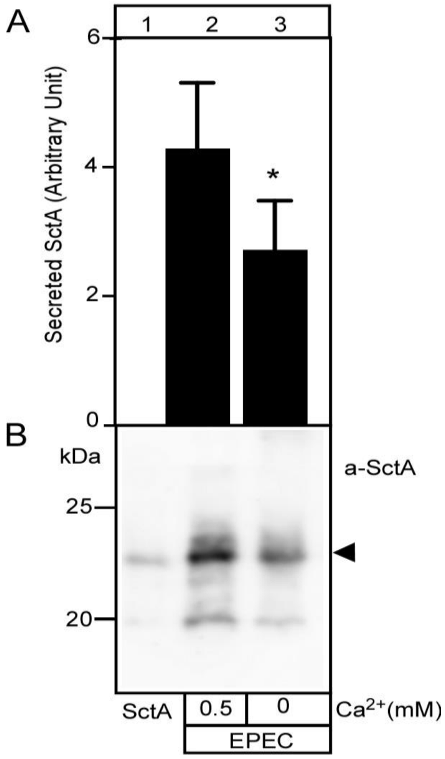

Fig. S4: Secretion of SctA in EPEC in absence and presence of Ca^{2+} (Related to Fig. 3)

A and B) SctA secretion is affected by Ca^{2+} concentrations. SctA secretion was monitored in high or low Ca^{2+} containing medium by immuno-staining using antibodies against SctA. **A)** Quantification of SctA signal intensities was performed using Image J software (Schneider *et al.,* 2012) and shown in bar graphs with SEM; *n = 3* biological repeats.

B) A representative image of immuno-detection showing SctA secretion and used for quantification in **A** is shown; *n = 3* biological repeats.

Supplementary Tables

Table S1: Genetic constructs

Gene	Uniprot accession	Plasmid name	Vector	Source
sctA-phoA	B7UM94, P00634	pLMB2059	pBAD501	This study
phoA	P00634	pIMBB953	pBAD501	Gouridis *et al,* 2010
prophoA	P00634	pIMBB882	pBAD501	Gouridis *et al,* 2010
His-*sctW*	B7UM95	pIMBB1305	pASK IBA 7^{+}	Portaliou *et al*., 2017
His-*sctW*-N1 (V14A-F15A-N16A-S19A-L20A)	B7UM95	pLMB1780	pASK IBA 7^{+}	This study
His-*sctW*-N2 (L41A-I42A-N43A-L44A-Q45A-N46A)	B7UM95	pLMB1761	pASK IBA 7^{+}	This study
His-*sctW (R333D)*	B7UM95	pIMBB1543	pASK IBA 7^{+}	Portaliou *et al.,* 2017
His-*sctW (N1-278)*	B7UM95	pLMB0089	pASK IBA 7^{+}	Portaliou *et al.,* 2017

Supplementary Materials

Table S2: Media composition

Medium	Composition	Source
M9-mod1	33.7 mM Na_2HPO_4; 22 mM KH_2PO_4; 8.55 mM NaCl; 9.35 mM NH_4Cl; 0.4% w/v Glucose; 0.2% w/v casamino acids; 5mM $MgSO_4$ and 0.5 mM $CaCl_2$	Biao *et al.,* 2018
M9-mod2	50mM HEPES, 8.55 mM NaCl, 9.35 mM NH_4Cl, 0.4% w/v Glycerol, 0.4% w/v casamino acids, 5 mM $MgSO_4$ and 0.5 mM $CaCl_2$.	This study

Antisera

Rabbit polyclonal antibodies against the indicated purified proteins were raised by Davids Biotechnologie (Germany). T3SS antibodies were further purified by negative immuno-absorption, using membranes isolated from EPEC strains that lacked the gene of interest, *i.e.* for α-SctW, membranes isolated from EPECΔ*sctW* cells were used.

Table S3: Antisera

**Antisera**	**Animal source**	**Reference or commercial source**
α-PhoA	Rabbit	Gouridis *et al,* 2009
α-SctA	Rabbit	Chen *et al,* 2011; Creasey et al, 2003
α-SctW	Rabbit	Portaliou *et al.,* 2017
α-Rabbit	Goat	Jackson Immuno Research Europe Ltd.

Bacterial strains

EPEC knock-out strains were generated following Datsenko and Wanner protocol (Datsenko *et al.,* 2000).

Table S4: Bacterial strains

***E. coli* strains**	**Description**	**Reference**
DH5α	F⁻ *endA1 glnV44 thi-1 recA1 relA1 gyrA96 deoR nupG purB20* φ80d*lacZ*ΔM15 Δ(*lacZYA-argF*)U169, hsdR17 ($r_K^-m_K^+$), λ⁻	Invitrogen
BL-21(DE)	*E. coli* str. B F⁻ *ompT gal dcm lon hsdS*$_B$(r_B^- m_B^-) λ(DE3 [*lacI lacUV5-T7p07 ind1 sam7 nin5*]) [*malB*$^+$]$_{K-12}$(λ^S)	Studier *et al.,* 1990
EPEC	*E. coli* O127:H6 (strain E2348/69)	Levine *et al.,* 1978
EPECΔ*sctL*	Δ*escL::* nptII(Kan^R)	This study
EPECΔ*sctO*	Δ*escO::* nptII(Kan^R)	This study
EPECΔ*sctN*	Δ*escN::* nptII(Kan^R)	Iguchi et *al.,* 2009
EPECΔ*sctU*	Δ*sctU*:: *nptII*(Kan^R)	This study
EPECΔ*sctV*	Δ*sctV*:: *nptII*(Kan^R)	Portaliou *et al.,* 2017
EPECΔ*escP*	Δ*sctP*:: *nptII*(Kan^R)	This study
EPECΔ*escI*	Δ*sctI*:: *nptII*(Kan^R)	This study
EPECΔ*sctW*	Δ*sctW*:: nptII(Kan^R)	Munera *et al.,* 2010
EPECΔ*sepD*	Δ*sepD*:: nptII(Kan^R)	Iguchi *et al.,* 2009
EPECΔ*cesL*	Δ*cesL::*nptII(Kan^R)	Portaliou *et al.,* 2017
EPECΔ*sctA*	Δ*sctA::*nptII(Kan^R)	This study
EPECΔ*cesAB*	Δ*cesAB*::nptII(Kan^R)	Iguchi *et al.*, 2009

Table S5: Vectors

**Vector**	**Antibiotic resistance**	**Origin of replication**	**Promoter**	**Reference**
pBAD501PhoA	Gentamicin	p15A	pAra	Chatzi *et al*., 2017
pASK-IBA7$^+$	Ampicillin	pBR322	pTet	IBA life sciences;

				(Guzman *et al.*, 1995)

Table S6: List of primers

Primer name	Forward/ Reverse	Gene cloned/ deleted	Sequence (5' to 3') (restriction sites underlined)
X258	For	*sctA*	GGGAATTCCATATGGATACATCAACTACAGCA
X2288	Rev		CCCAAGCTTTTTACCAAGGGATATTCCTGAAATAGTT
X1467	For	*sctL*	AGTCAAATATCTTTTACCGAAAAGTGGAGAAATAAAACCAACTCATATTATAGGCTGGAGCTGCTTC
X1468	Rev		TAATTAAAAATATTGGCTGTGAGCCAATGGTCATTAATTGAGACATATCACATATGAATATCCTCCTTAG
X1518	For	*sctO*	AGTAGTTACGAAAAAACGATTGAAAGCCTATTCAAAGTGGTTGCCTGAGTTAGGCTGGAGCTGCTTC
X1519	Rev		CTGATGGCCGAAAAGAAACAGGCTCTATCAAATTTCTTTTTAGAGAAACTCATATGAATATCCTCCTTAG
X1705	For	*sctP*	TTTCTCTAAAAAGAAATTTGATAGAGCCTGTTTCTTTTCGGCCATCAGATTAGGCTGGAGCTGCTTC
X1706	Rev		ACATAGTCTTTTTTATGATATAAAAAAACATGATTTCTATTATTTTGGCTCATATGAATATCCTCCTTAG
X2231	For	*sctA*	TTTTTTTATAGTTTTTGTCATGCTAAGAAAGATTATGAAGAGGTATATACTAGGCTGGAGCTGCTTC
X2232	Rev		TTATTTACCAAGGGATATTCCTGAAATAGTTCTATATTGCAGTGACTGCACATATGAATATCCTCCTTAG

SctA-PhoA sequence

The complete SctA-PhoA aminoacyl residue sequence (residue numbering from original PhoA sequence was maintained)

```
        10         20         30         40         50         60         70
MDTSTTASVA SANASTSTSM AYDLGSMSKD DVIDLFNKLG VFQAAILMFA YMYQAQSDLS IAKFADMNEA
        80         90        100        110        120        130        140
SKESTTAQKM ANLVDAKIAD VQSSSDKNAK AQLPDEVISY INDPRNDITI SGIDNINAQL GAGDLQTVKA
       150        160        170        180        190192         30         40
AISAKANNLT TTVNNSQLEI QQMSNTLNLL TSARSDMQSL QYRTISGISL GKRTPEMPVLE NRAAQGDITA
        50         60         70         80         90        100        110
PGGARRLTGD QTAALRDSLS DKPAKNIILL IGDGMGDSEI TAARNYAEGA GGFFKGIDAL PLTGQYTHYA
       120        130        140        150        160        170        180
LNKKTGKPDY VTDSAASATA WSTGVKTYNG ALGVDIHEKD HPTILEMAKA AGLATGNVST AELQDATPAA
       190        200        210        220        230        240        250
LVAHVTSRKC YGPSATSEKC PGNALEKGGK GSITEQLLNA RADVTLGGGA KTFAETATAG EWQGKTLREQ
       260        270        280        290        300        310        320
AQARGYQLVS DAASLNSVTE ANQQKPLLGL FADGNMPVRW LGPKATYHGN IDKPAVTCTP NPQRNDSVPT
       330        340        350        360        370        380        390
LAQMTDKAIE LLSKNEKGFF LQVEGASIDK QDHAANPCGQ IGETVDLDEA VQRALEFAKK EGNTLVIVTA
       400        410        420        430        440        450        460
DHAHASQIVA PDTKAPGLTQ ALNTKDGAVM VMSYGNSEED SQEHTGSQLR IAAYGPHAAN VVGLTDQTDL
       470
FYTMKAALGL K
```

Reference:

B. Yuan, A. Economou, and S. Karamanou, “Optimization of type 3 protein secretion in enteropathogenic Escherichia coli,” FEMS Microbiol. Lett., vol. 365, no. 14, pp. 1–7, 2018.

Chatzi, K.E., Sardis, M.F., Tsirigotaki, A., Koukaki, M., Sostaric, N., Konijnenberg, A., Sobott, F., Kalodimos, C.G., Karamanou, S., and Economou, A. Preprotein mature domains contain translocase targeting signals that are essential for secretion. J Cell Biol 2017; 216: 1357-1369.

Chen, L., Balabanidou, V., Remeta, D.P., Minetti, C.A., Portaliou, A.G., Economou, A., and Kalodimos, C.G. Structural instability tuning as a regulatory mechanism in protein-protein interactions. Molecular cell 2011; 44: 734-744.

Creasey, E.A., Friedberg, D., Shaw, R.K., Umanski, T., Knutton, S., Rosenshine, I., and Frankel, G. CesAB is an enteropathogenic Escherichia coli chaperone for the type-III translocator proteins EspA and EspB. Microbiology 2003; 149: 3639-3647.

Datsenko, K.A. and B.L. Wanner, One-step inactivation of chromosomal genes in Escherichia coli K-12 using PCR products. 2000. 97(12): p. 6640-6645.

Gouridis G, Karamanou S, Gelis I, Kalodimos CG, Economou A (2009) Signal peptides are allosteric activators of the protein translocase. Nature 462: 363-367.

Gouridis, G., Karamanou, S., Koukaki, M., Economou A. (2010). *In vitro* assays to analyze translocation of the model secretory preprotein alkaline phosphatase. Methods Mol Biol, 2010. 619: p. 157-72.

Guzman, L.M., Belin, D., Carson, M.J., and Beckwith, J. Tight regulation, modulation, and high-level expression by vectors containing the arabinose PBAD promoter. J Bacteriol 1995; 177: 4121-4130.

Iguchi A, Thomson NR, Ogura Y, Saunders D, Ooka T, Henderson IR, Harris D, Asadulghani M, Kurokawa K, Dean P, Kenny B, Quail MA, Thurston S, Dougan G, Hayashi T, Parkhill J, Frankel G (2009) Complete genome sequence and comparative genome analysis of enteropathogenic Escherichia coli O127:H6 strain E2348/69. J Bacteriol 191: 347-354.

Levine, M.M., Bergquist, E.J., Nalin, D.R., Waterman, D.H., Hornick, R.B., Young, C.R., and Sotman, S. Escherichia coli strains that cause diarrhoea but do not produce heat-labile or heatstable enterotoxins and are non-invasive. Lancet 1978; 1: 1119-1122.

Munera D, Crepin VF, Marches O, Frankel G (2010) N-terminal type III secretion signal of enteropathogenic Escherichia coli translocator proteins. Journal of bacteriology 192: 3534- 3539.

Portaliou A. G. et al., “Hierarchical protein targeting and secretion is controlled by an affinity switch in the type III secretion system of enteropathogenic Escherichia coli,” EMBO J., vol. 36, no. 23, pp. 3517–3531, 2017.

Schneider, C.A., Rasband, W.S., and Eliceiri, K.W. NIH Image to ImageJ: 25 years of image analysis. Nature methods 2012; 9: 671.

Studier FW, Rosenberg AH, Dunn JJ, Dubendorff JW (1990) Use of T7 RNA polymerase to direct expression of cloned genes. Methods in enzymology 185: 60-89
